# Antiproliferative Activity of Combined Biochanin A and Ginsenoside Rh_2_ on MDA-MB-231 and MCF-7 Human Breast Cancer Cells

**DOI:** 10.3390/molecules23112908

**Published:** 2018-11-08

**Authors:** Guixing Ren, Zhenxing Shi, Cong Teng, Yang Yao

**Affiliations:** 1 College of Pharmacy and Biological Engineering, Chengdu University, No.1 Shilling Road, Chenglo Avenue, Longquan District, Chengdu 610106, China; guixin@126.com; 2 Laboratory of Biomass and Green Technologies, University of Liege-Gembloux Agro-Bio Tech, Passage des Déportés 2, B-5030 Gembloux, Belgium; zhenxing@126.com; 3 Institute of Crop Science, Chinese Academy of Agricultural Sciences, No.80 South Xueyuan Road, Haidian District, Beijing 100081, China; 82101172124@caas.cn

**Keywords:** synergy, biochanin A, Rh_2_, breast cancer, antiproliferative activity

## Abstract

Breast cancer is the most frequently diagnosed cancer in women worldwide. The antiproliferative activities of biochanin A (BA) and ginsenoside Rh_2_ were determined by evaluating their inhibitory effect on MDA-MB-231 human breast cancer cell proliferation. The combination of BA with Rh_2_ was also assessed. In MDA cells, combination treatment led to a decrease in the EC_50_ values of BA and Rh_2_ to 25.20 μM and 22.75 μM, respectively. In MCF-7 cells, the EC_50_ values of combined BA and Rh_2_ decreased to 27.68 μM and 25.41 μM, respectively. BA combined with Rh_2_ also improved the inhibition of MDA-MB-231 and MCF-7 cell migration and invasion compared to the individual compounds. Western blot analysis demonstrated upregulation in p-p53, p-p38, and p-ASK1 proteins while levels of TRAF2 were downregulated. These results suggest that BA combined with Rh_2_ exhibits synergistic effects against MDA-MB-231 and MCF-7 cell proliferation.

## 1. Introduction

Today, breast cancer is the second leading cause of mortality in women worldwide. More than two hundred thousand new breast cancer cases were diagnosed in the United States in 2016 according to epidemiology, surveillance, and the end result program seer.cancer.gov [1]. Triple negative breast cancer is characterized by tumors that are human epidermal growth factor receptor 2-negative, progesterone receptor-negative, and estrogen receptor-negative [2]. MDA-MB-231 and MCF-7 human cancer cell lines are estrogen receptor and estrogen receptor-positive cells, respectively. These cell lines are well-established in vitro models for evaluating estrogen-responsive or estrogen-independent antineoplastic drugs [3].

Numerous studies have shown that natural products play an important role in the inhibition and therapy of cancers [4]. The nutritional function of food shows the synergistic and additive effects of phytochemicals compared to a single compound [5]. Differences in solubility, polarity, and molecular size of these compounds can affect their distribution and bioavailability in various organs, tissues, cells, and subcellular organelles. Furthermore, purified phytochemicals may lose some of their bioactivity and behave differently than in whole foods [6]. Similarly, chemotherapeutic combinatorial methods have been conducted to decrease drug side effects, slow the growth of cancer cells, and achieve results superior to those of one active drug alone.

Biochanin A (BA), an isoflavone, has been shown to possess antiviral [7], antioxidant [8], anticarcinogenic [9], anti-inflammatory [10], and protective effects on endothelial integrity and function [11]. BA exhibits promise as a phytochemical driving the inhibition of breast cancer through promoting estrogen receptor-positive cell proliferation [12]. Rh_2_ (protopanaxadiol-type) is the major dammarane-type saponin ginsenoside. Previous studies have shown that Rh_2_ has beneficial impacts against breast cancer [13], hepatoma cells [14], glioma cells [15], prostate cancer cells [16], and lung cancer cells [17]. Previous publications describe the interactions between isoflavone and ginseng saponins and their role in suppressing MDA-MB-231 cell proliferation. The aim of this study was to determine whether BA and Rh2 have additive and/or synergistic effects on MDA-MB-231 and MCF-7 human breast cancer cell proliferation.

## 2. Results and Discussion

### 2.1. Cytotoxicity and Antiproliferative Activities of MDA-MB-231 and MCF-7

**Figure 1:**
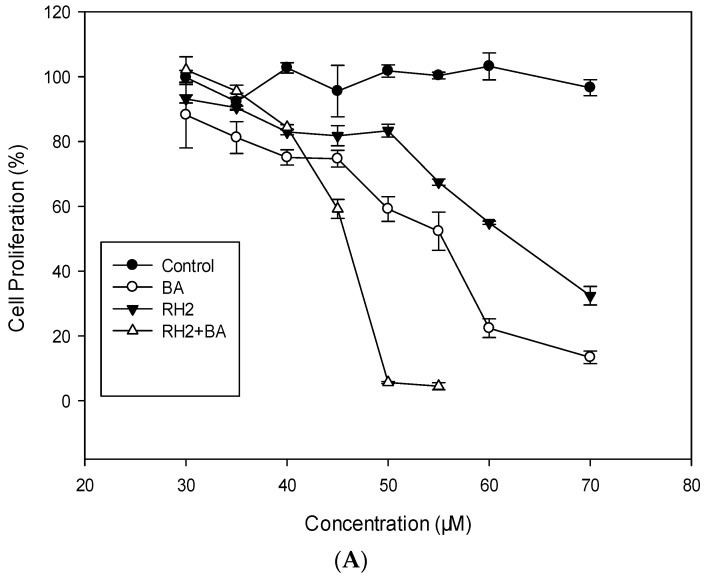

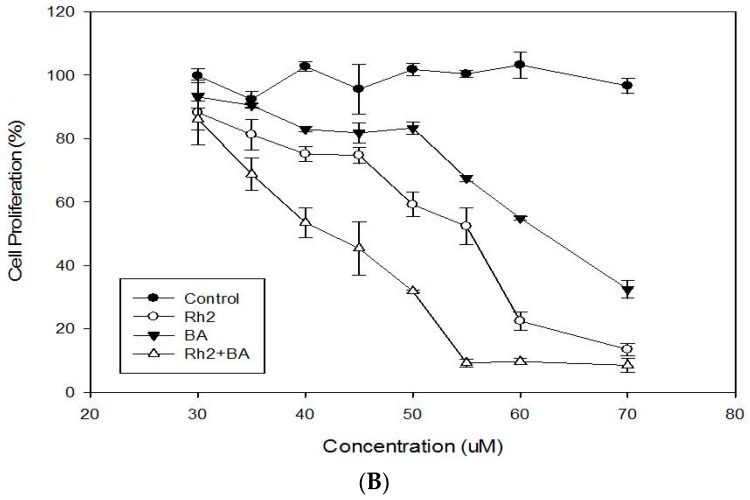
Antiproliferative effects of BA, Rh_2_, and the combination of BA with Rh_2_ on MDA (**A**) and MCF-7 (**B**) human breast cancer cell lines (mean ± SD, *n* = 3). Each value represents the mean ± SD of triplicate biological experiments.

BA and Rh_2_ both show no cytotoxicity at doses of 10–100 µM (data not shown). These data is supported by previous work performed by Tan and Kim reporting that 100 μM BA had no toxic effects in PC12 cells [18]. Furthermore, Quan’s studies have shown that Rh_2_ at 80 µM dose exhibited no cytotoxic activity in the cells [19]. The antiproliferative effects of BA, Rh_2_, and the combination of BA with Rh_2_ on cell growth are presented in Figure 1. BA, at doses of 30–70 μM (*p* < 0.05), shows dose-dependent antiproliferative effects on MDA-MB-231 and MCF-7 cell growth (Figure 1A,B). The EC_50_ values of BA in inhibiting the growth of MDA-MB-231 and MCF-7 were 63.76 μM and 59.76 μM, respectively. Rh_2_ also showed a dose-dependent prevention of proliferation in both cell lines at doses of 30–70 μM (Figure 1A,B). The EC_50_ values of Rh_2_ in inhibiting MDA-MB-231 and MCF-7 cell line proliferation were 57.53 μM and 52.53 μM, respectively (Table 1).

Tsu et al. reported that BA concentrations exceeding 30 μg/mL in MCF-7 cells exhibited dose-dependent effects on cell proliferation. At 100 μg/mL, MCF-7 cell proliferation was attenuated by approximately 80% [20]. Moon et al. demonstrated a role for BA in breast cancer prevention using xenograft mouse models. In this study, BA treatment groups (5 mg/kg) had significantly inhibited growth of tumors compared to the control group [21]. Choi et al. showed that Rh_2_ treatment inhibited the viability of MDA-MB-231 cells by 28% and 85% at doses of 40 μM and 60 μM, respectively [22].

Drug combinations have received increasing attention due to the advantages of lower drug doses, reduced side effects, and improved anticancer effects. Data in Figure 1 suggest that the combination of Rh_2_ plus BA significantly increased the antiproliferative activity of cells compared to the effects of Rh_2_ or BA alone. In MDA-MB-231 cells, the EC_50_ values of combined BA and Rh_2_ were decreased to 25.20 μM and 22.75 μM, respectively, which represents 2.53-fold and 2.52-fold less than those of BA and Rh_2_ alone. In MCF-7 cells, the EC_50_ values of combined BA and Rh_2_ were decreased to 27.68 μM and 25.41 μM, respectively, which were 2.16-fold and 2.07-fold less than those of BA and Rh_2_ alone. At 50, 75, 90, and 95% inhibition of cell growth, the CI numbers of the combined BA and Rh_2_ treatment were 0.42 ± 0.05, 0.55 ± 0.09, 0.72 ± 0.11, and 0.88 ± 0.10, respectively, suggesting that considerable synergistic effects exist at all tested doses (Table 2).

Moon et al. compared the co-administration of quercetin, epigallocatechin-3-gallate, and BA with the administration of BA alone in a murine xenograft model. The results showed that combined BA, quercetin, and epigallocatechin-3-gallate (5 mg/kg) led to improved efficacy comparable to that of 15 mg/kg BA alone [21]. This could be in part due to the fact that the combination of BA, quercetin, and epigallocatechin-3-gallate causes improved oral bioavailability of BA in animal models. The combination significantly increases the level of BA in plasma samples after either oral administration or intravenous injection. Oral bioavailability was improved three-fold compared to the administration of BA alone [23]. Xie et al. evaluated the synergistic effect of Rh_2_ plus paclitaxel on LNCaP prostate cancer models both in vitro and in vivo. The data suggested that Rh_2_ plus paclitaxel exhibit synergy in LNCaP cells at less than 50% of the effective dose values. The combination of Rh_2_ and paclitaxel resulted in a significant reduction of prostate specific antigen in serum, and inhibition in the growth of LNCaP tumors. In addition, immunohistochemistry results showed obvious effects on proliferation agents [24].

### 2.2. Enhanced Inhibition of Cell Migration

**Figure 2:**
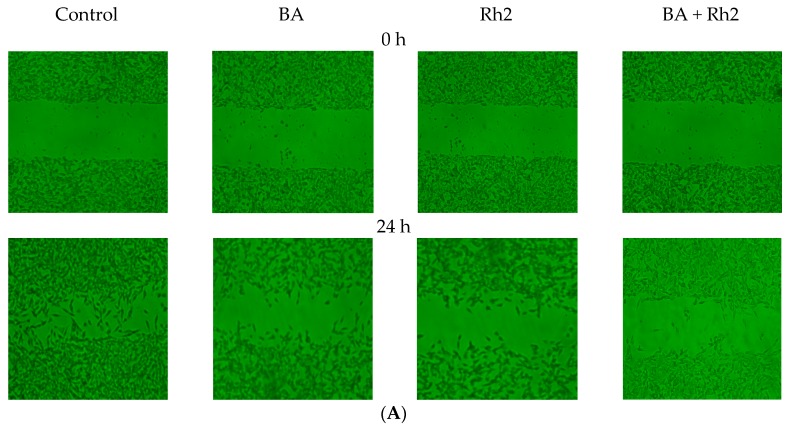

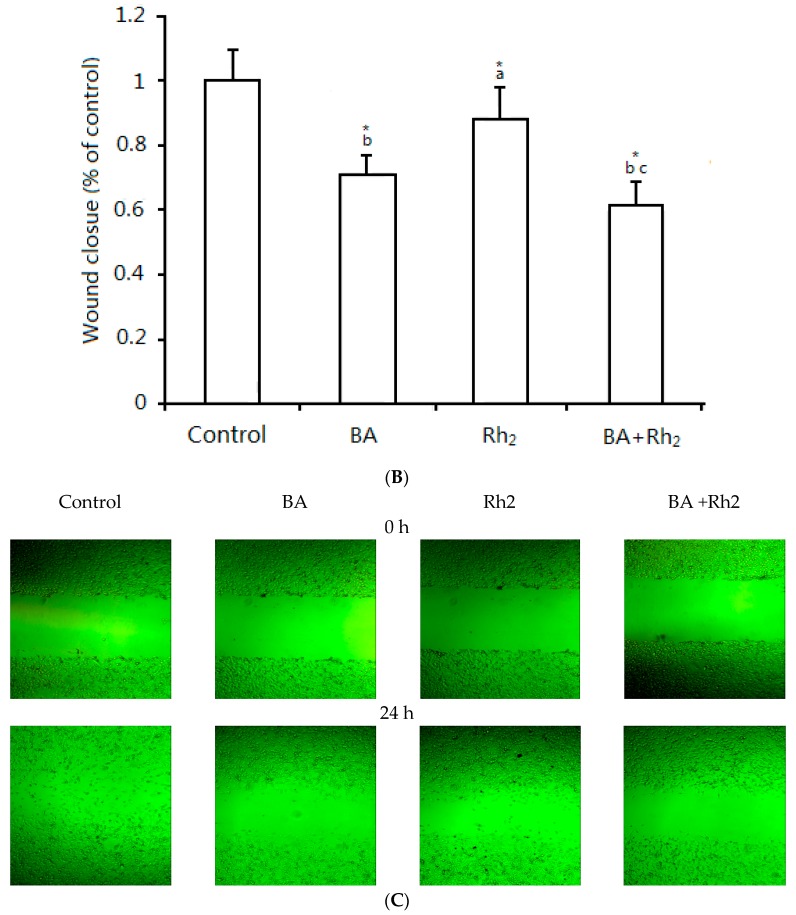

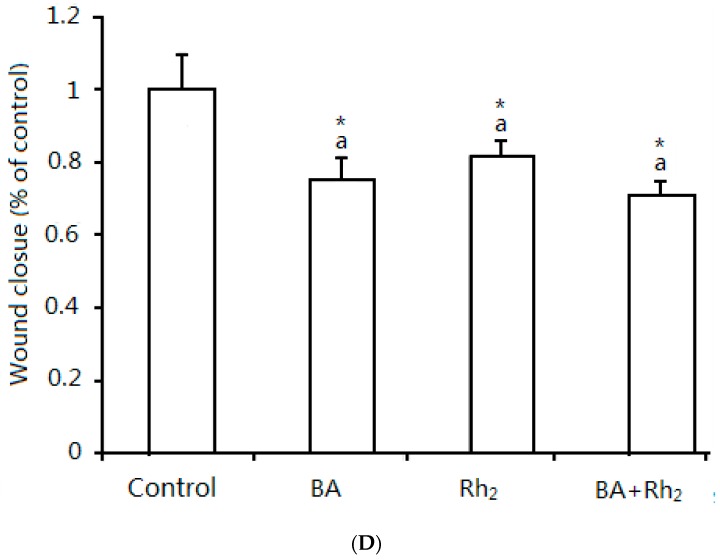
For the scratch assay, wounds were made when MDA (**A**,**B**) and MCF-7 (**C**,**D**) cells were 90–100% confluent and after an overnight starvation. Cells were treated with vehicle control, 63.76 μM BA, 57.53 μM Rh_2_, or 25.20 μM BA + 22.75 μM Rh_2_ for 24 h. The closure of wounds was imaged and measured at 0 and 24 h. An asterisk (*) indicates a significant difference from the control (*p* < 0.05). * Compared to the control, *p* < 0.05. Different letters showed significant difference in sample groups (*p* < 0.05).

Tumor cell migration and invasion are the two most important traits of metastasis. To further study the inhibitory roles of BA, Rh_2_, and BA combined with Rh_2_ on metastasis, we evaluated their effect on cell migration and invasion. A wound-healing assay was used to evaluate the inhibition of BA, Rh_2_, and BA with Rh2 on cell migration. Both BA and Rh_2_ inhibited wound closure in MDA-MB-231 and MCF-7 cells (Figure 2A,C). In MDA cells, the combination of BA plus Rh_2_ significantly enhanced the inhibition compared to Rh_2_ alone. The combination treatment inhibited wound closure by 39% and 29% compared to that of the control in MDA-MB-231 and MCF-7 cells, respectively (Figure 2B,D).

**Figure 3:**
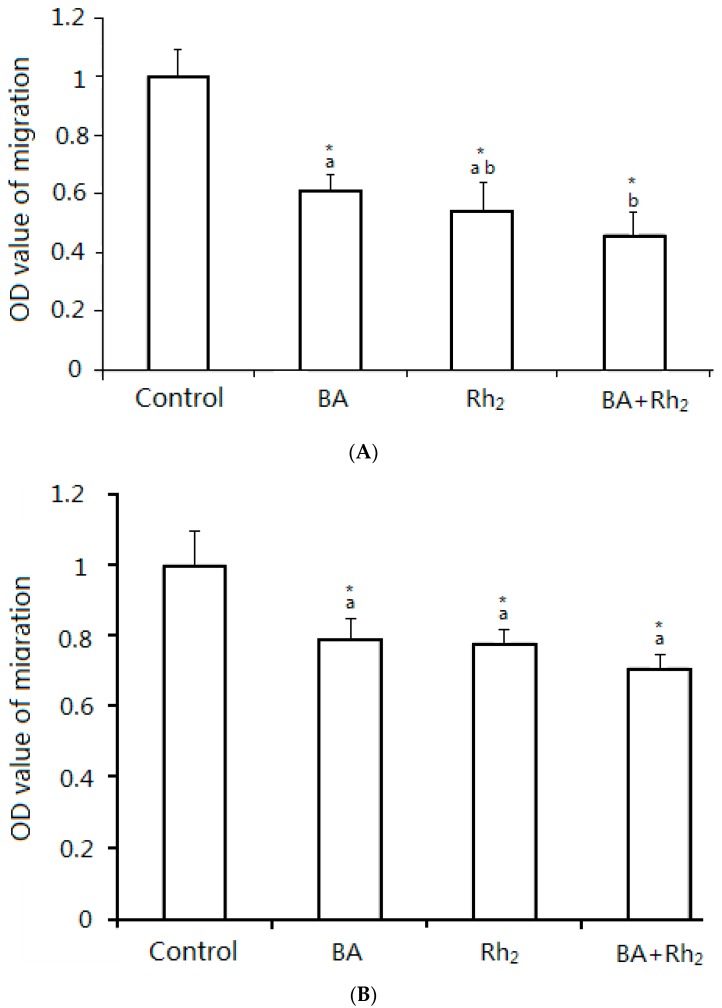
In the trans-well chamber assay, MDA (**A**) and MCF-7 (**B**) cells were treated with vehicle control, EC_50_ values of BA, Rh_2_ and BA with Rh_2_ for 48 h. Cells suspended in serum-free media were seeded on the upper membrane of the trans-well chamber and incubated for 48 h. Complete growth medium was added on the bottom. Cells on the lower membrane of the chambers were counted. Data are presented as mean ± SD. An asterisk (*) indicates a significant difference from the control (*p* < 0.05). * Compared to the control, *p* < 0.05. Different letters showed significant difference in sample groups (*p* < 0.05).

To measure the effect of BA, Rh_2_, and BA plus Rh_2_ on cell invasion we used a trans-well chamber assay. The mixture of BA plus Rh_2_ increased the inhibition in both cell lines compared to BA or Rh_2_ alone (Figure 3A,B). In MDA cells, the BA plus Rh_2_ significantly increased inhibition compared to BA. Migration of MDA-MB-231 and MCF-7 cells was separately inhibited by 57% and 29% under combined BA and Rh_2_ treatment compared to the control. In summary, these results indicate that BA, Rh_2_, and BA plus Rh_2_ exert strong inhibition activities on the migration and invasion of triple-negative breast cancer cells. Additionally, the combination exhibited greater effectiveness in these two assays.

Xiao et al. showed that BA exhibits anticancer effects by evaluating the migratory effect of BA on wound healing and invasion in SK-Mel-28 human malignant melanoma cells. They observe that treatment with 0, 10, 50 and 100 µM doses of BA result in the suppression of migration and invasion in a dose-dependent manner [25].

### 2.3. Modulations of Protein Expression and Signalling Pathways

**Figure 4:**
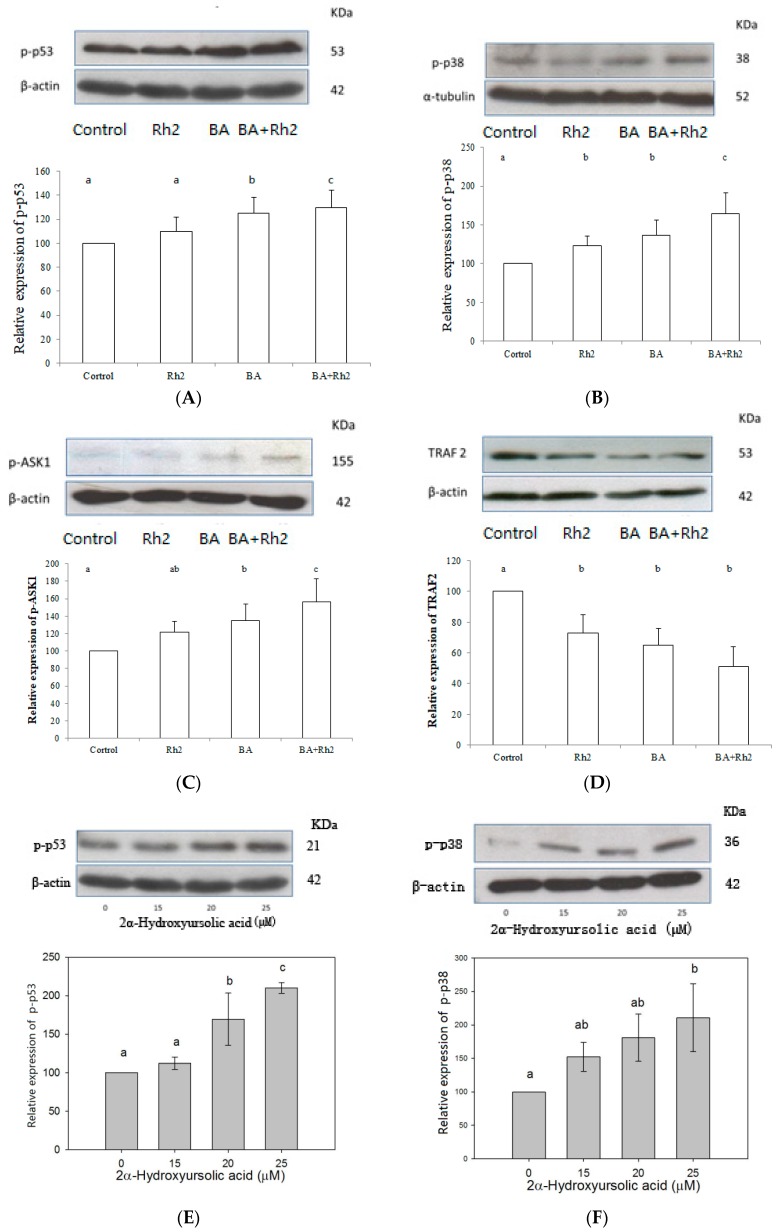

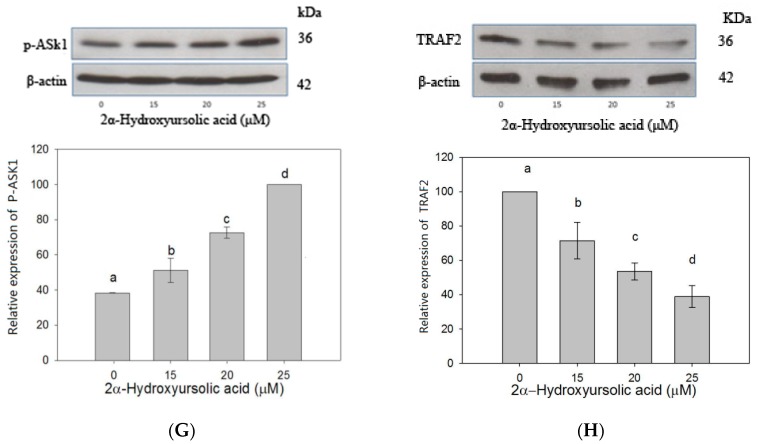
Effects of BA, Rh_2_ and the combination of BA and Rh_2_ on expression of p-p53 (**A**,**E**), p-p38 (**B**,**F**), p-ASK1 (**C**,**G**), and TRAF 2 (**D**,**H**) in MDA-MB-231 and MCF-7 human breast cancer cells. Bars with no letters in common are significantly different (*p* < 0.05). Each value represents the mean ± SD of triplicates. Different letters showed significant difference in sample groups (*p* < 0.05).

The regulation of protein expression and signaling pathways in MDA-MB-231 and MCF-7 cells were similar (Figure 4). BA demonstrated considerable capabilities in the upregulation of phosphorylated p53 (p-p53) and phosphorylated p38 (p-p38) protein levels relative to Rh_2_ (Figure 4A,B,E,F). The inhibitory effects were further improved by BA and Rh_2_ combination. Cell growth, apoptosis and cycle progression are regulated by p38 MAPK [26]. Expression of phosphorylated apoptosis signal-regulating kinase 1 (p-ASK1) was significantly enhanced after treatment with the BA plus Rh_2_ combination compared to the control (Figure 4C,G). However, the combination of BA and Rh_2_ significantly downregulated the protein expression of TNF receptor associated factor 2 (TRAF2), which serves as a mediator of the anti-apoptotic marker (Figure 4D,H). Upregulated p-ASK1 and downregulated TRAF2 promote the kinase p38 pathway, resulting in the phosphorylation of p53 and thus triggering anti-proliferation and apoptosis in cells [27].

Liu et al. evaluated the proliferative effect of Rh_2_ in KG1-α and K562 human leukemia cells in vitro and the inhibitory effect on the growth of human leukemia xenograft tumors in vivo. The data showed that Rh_2_ exerts antiproliferative effects on those cells by increasing histone acetylation. In addition, Rh_2_ significantly modulated JNK, p-JNK, p38, and p-p38 protein expression thus inducing apoptosis by activating the MAPK signaling pathway [28]. Choi et al. observed that Rh_2_ inhibits MDA-MB-231 cell viability by reducing the contents of phosphorylated retinoblastoma protein and lowering the transcriptional activity of E2 promoter binding factor 1, as shown by the luciferase reporter assay. In addition, Rh_2_ regulated cyclin-dependent kinases (Cdk), cyclins, and the cell cycle, resulting in induced interaction between Cdk4/Cdk6 and cyclin D1, as well as improved recruitment of p15Ink4B and p27Kip1 to cyclin D1/Cdk4 and cyclin D1/Cdk6 complexes [22]. It has also been reported that Rh_2_ induces apoptotic cell death by triggering caspase-1 and caspase-3, and upregulating Bax in neuroblastoma cells [29]. Ginsenoside Rg5 promotes breast cancer cell apoptosis by inducing G0/G1 cell cycle arrest in MCF-7 and MDA-MB-453 human breast cancer cell lines. P53-dependent apoptosis indicates that the tumor inhibitor p53 induces cell self-destruction through the endogenous mitochondrial and exogenous death receptor pathways [30]. Thus, p53-dependent apoptosis could be used to lead to the expression of proapoptotic members. If cells undergo DNA damage, p53 arrests the cell cycle by p21 or by induction of apoptosis. To respond to DNA damage or cellular stress, p53 is stabilized by post-transcriptional modifications, and the concentration of p53 increases [31]. Stabilization and activation of p53 is responsible for cellular antiproliferative mechanisms, such as growth arrest, cell senescence, and apoptosis [32].

## 3. Materials and Methods

### 3.1. Chemicals

Rh_2_ and BA were purchased from the National Institutes for Food and Drug Control (Beijing, China). MDA-MB-231 human breast cancer cells were purchased from the American Type Culture Collection (Manassas, VA, USA). Fetal bovine serum, α-minimum essential medium (α-MEM), Hank’s Balanced Salt Solution (HBSS), 2-(4-(2-hydroxyethyl)-1-piperazinyl)-ethanesulfonic acid (HEPES) and phosphate-buffered saline (PBS) were purchased from Gibco Life Technologies (Grand Island, NY, USA). Methylene blue and dimethyl sulfoxide (DMSO) were purchased from Sigma-Aldrich (St. Louis, MO, USA). Extracellular matrix (ECM) invasion assay kits were purchased from Millipore (Billerica, MA, USA).

### 3.2. Cytotoxicity Activity

The cytotoxicity of Rh_2_ or BA towards MDA-MB-231 cells and MCF-7 cells was evaluated by methylene blue assay as reported previously [33]. In brief, MDA-MB-231 cells were cultured in α-MEM containing 10 mM HEPES, 1% antibiotic-antimycotic and 10% fetal bovine serum. MCF-7 cells were maintained in α-MEM containing 10 mM HEPES, 1% antibiotic-antimycotic, 0.01 mg/mL insulin, and 10% fetal bovine serum as described previously [34]. MDA-MB-231 and MCF-7 cells were maintained in an incubator at 5% CO_2_ and 37 °C. A total of 5 × 104 cancer cells in growth media were placed in each well of a 96-well flat-bottom plate. After that, the growth medium was changed to include the treatments or 100 µL new medium as control. After 24 h of incubation, cells were rinsed with phosphate-buffered saline. Cells were then stained with methylene blue solution (0.6% methylene blue, 0.67% glutaraldehyde, and 98% HBSS) and incubated for 1 h. The solution was removed and washed with deionized water. After the wells were dried, methylene blue was stained in cells with the elution buffer (50% ethanol, 49% PBS, and 1% acetic acid) and rotated for 15 min. The absorbance was read at 570 nm by using a microplate reader (Bio-Rad, Danvers, MA, USA). Cytotoxicity was evaluated as a percentage compared to the control.

### 3.3. Antiproliferative Activity

The antiproliferative activities of Rh_2_ and BA towards MDA-MB-231 cells and MCF-7 cells were measured by the methylene blue assay [24]. MDA-MB-231 cells and MCF-7 cells were incubated in the same conditions described above. The cells were seeded at 2.5 × 10^6^ cells/mL, and various concentrations of Rh_2_, BA, or control were added to the cells. After 72 h, cell proliferation was determined using the methylene blue assay measuring absorbance at 570 nm.

### 3.4. Combination Study

A study on the combination of Rh2 and BA towards MDA-MB-231 and MCF-7 cell proliferation was designed. The EC_50_ values of Rh_2_ and BA were evaluated according to dose-response curves. The combination concentrations of Rh_2_ and BA were 0.125 × EC_50_, 0.25 × EC_50_, 0.50 × EC_50_, 0.75 × EC_50_, 1.00 × EC_50_, and 1.25 × EC_50_, respectively. Finally, a series of concentrations of Rh_2_ and BA were mixed to generate the dose-response curve in the MDA-MB-231and MCF-7 cell proliferation models. A combination index (CI) was calculated for the combinations of Rh_2_ and BA using the Compu Syn software (ComboSyn, Inc., Paramus, NJ, USA), on account of the mass-action law and Chou–Talalay equation [35]. A value of CI below 1 indicates a synergistic effect of a combination, equal to 1 shows additive effects, and higher than 1 indicates antagonistic effects.

### 3.5. Wound-Healing Assay

A wound closure seeding model was built using silicon culture inserts (Ibidi, LLC, Munchen, Germany) with two individual wells for cell seeding. The insert was placed in a culture dish, and 5 × 10^5^ cells/mL of MDA-MB-231 and MCF-7 cells were plated in each well and grown to form a confluent and homogeneous layer. The culture insert was removed, and a cell-free area was recorded after 24 h cell seeding. The wound was approximately 500 μM wide. Healing of the wound by migrating cells after Rh_2_, BA or Rh_2_ plus BA treatment was observed after 24 h by light microscopy (CX-2, Olympus) and analyzed using Image J software (NIH, Bethesda, Maryland, USA) [36].

### 3.6. Invasion Assay

An ECM kit assay was used to evaluate cell invasiveness. According to the manufacturer’s protocols, 5 × 10^5^ cells were suspended in 300 μL of serum-free media and plated on an ECM-coated membrane insert. The invasion assay was examined after 48 h of control, Rh_2_, BA or Rh2 combined with BA treatment. After that, the cells in the upper insert were wiped away, while the cells on the lower side were stained [37].

### 3.7. Western Blot Analysis

Western blot analysis was conducted according to the method of Yao et al. [38]. The supernatant was collected after lysed cells were centrifuged at 12,000 rpm at 4 °C for 15 min. Protein extracts were separated with SDS-PAGE and transferred to PVDF membrane. After blocking, p-p53, p-p38, p-ASK1, TRAF2, and β-actin antibodies (Santa Cruz Biotechnology, Santa Cruz, CA, USA) were added and incubated. Membranes were washed and incubated in PBST and HRP-conjugated secondary antibody, respectively. The signals were detected using the Super Signal ELISA Pico Chemiluminescent Substrate (Thermo Fisher Scientific, Waltham, MA, USA).

### 3.8. Statistical Analysis

Data were analyzed using Sigma Plot software version 11.0 (Systat Software, Inc., Chicago, IL, USA). All values were expressed as the means ± SD of at least three independently performed experiments. Statistical analyses were conducted with Student’s *t*-test and analysis of variance (ANOVA) by SPSS software version 16.0. Differences with *p* < 0.05 were considered to be statistically significant.

## 4. Conclusions

In summary, a novel combinatorial treatment with BA plus Rh2 synergistically enhanced the antiproliferative effect in MDA-MB-231 and MCF-7 cells and was associated with the upregulated expression of p-p53, p-p38, and p-ASK1 and downregulated expression of TRAF2. Further in vivo studies are necessary to verify the efficacy and appropriate doses of the combination for alleviating receptor 2-negative, progesterone receptor-negative, and estrogen receptor-negative breast cancer in clinical trials.

## Figures and Tables

**Table 1:** The EC_50_ values of BA and Rh_2_ towards MDA-MB-231 and MCF-7 cells.

Component	EC_50_ Value			
	MDA-MB-231		MCF-7	
	Single	Combined	Single	Combined
Biochanin A	63.76 μM	25.20 μM	59.76 μM	27.68 μM
Rh2	57.53 μM	22.75 μM	52.53 μM	25.41 μM

**Table 2:** The CI numbers of combined BA and Rh_2_.

CI Values at Different Inhibition of Rates			
50%	75%	90%	95%
0.435	0.553	0.723	0.882
